# A systematic review of strategies used for controlling consumer moral hazard in health systems

**DOI:** 10.1186/s12913-022-08613-y

**Published:** 2022-10-18

**Authors:** Zohreh Koohi Rostamkalaee, Mehdi Jafari, Hasan Abolghasem Gorji

**Affiliations:** 1 Department of Health Services Management, School of Health Management and Information Sciences, Iran University of Medical Sciences, Tehran, Iran; 2 Health Management Research Institute, Iran University of Medical Sciences, Tehran, Iran

**Keywords:** Consumer Moral hazard, Health system, Systematic review

## Abstract

**Background:**

Consumer moral hazard refers to an increase in demand for health services or a decrease in preventive care due to insurance coverage. This phenomenon as one of the most evident forms of moral hazard must be reduced and prevented because of its important role in increasing health costs. This study aimed to determine and analyze the strategies used to control consumer moral hazards in health systems.

**Methods:**

In this systematic review. Web of Sciences, PubMed, Scopus, Embase, ProQuest, Iranian databases(Magiran and SID), and Google Scholar engine were searched using search terms related to moral hazard and healthcare utilization without time limitation. Eligible English and Persian studies on consumer moral hazard in health were included, and papers outside the health and in other languages were excluded. Thematic content analysis was used for data analysis.

**Results:**

Content analysis of 68 studies included in the study was presented in the form of two group, six themes, and 11 categories. Two group included “changing behavior at the time of receiving health services” and “changing behavior before needing health services.” The first group included four themes: demand-side cost sharing, health savings accounts, drug price regulation, and rationing of health services. The second approach consisted of two themes Development of incentive insurance programs and community empowerment.

**Conclusion:**

Strategies to control consumer moral hazards focus on changing consumer consumptive and health-related behaviors, which are designed according to the structure of health and financing systems. Since “changing consumptive behavior” strategies are the most commonly used strategies; therefore, it is necessary to strengthen strategies to control health-related behaviors and develop new strategies in future studies. In addition, in the application of existing strategies, the adaptation to the structure of the health and financing system, and the pattern of consumption of health services in society should be considered.

**Supplementary Information:**

The online version contains supplementary material available at 10.1186/s12913-022-08613-y.

## Background

Maximizing the health of people and populations is one of the main goals of any health system which leads to improving personal, social, and economic well-being [1]. The efficient function of the health system depends on providing improved health services at a minimum cost [2]. Evidence shows that health spending is growing faster than economic growth [1, 3]. On average, health spending in OECD countries was equivalent to 9% of GDP in 2018 [1]. The United States spent 18% of its GDP on health care in 2015 [4]. Moral hazard is one of the most important reasons for increasing health costs [5, 6]. Moral hazard is the change in health behavior and consumption of health services because of insurance coverage [7]. According to the theory of moral hazard, health insurance and third-party payers, by lowering the price of care, encourage the consumer to consume more care than when they consume at the market price [8]. Insurance coverage leads to the consumption of health services above an efficient level [9].

This phenomenon is classified in different ways: ex-ante moral hazard and ex-post moral hazard, hidden information and hidden action moral hazard, provider moral hazard, and consumer moral hazard [10, 11]. Ex-ante moral hazard occurs before illness and increases a person’s unhealthy behavior; in contrast, ex-post moral hazard occurs after the onset of illness and will increase costs by increasing demand and consuming unnecessary services. In the hidden information, the insurer cannot observe the real condition and severity of the disease to pay the cost according to the real need. In hidden action individuals’ precautionary measures are not visible, and the insured person does not take the necessary precautions to prevent the disease. Provider moral hazard occurs when a provider provides more services to increase its revenue. The provider moral hazard is also known as the provider’s induced demand. Consumer moral hazard means insured people demand more care than uninsured people [11, 12]. Additionally, consumer moral hazard deals with the reduction in preventive healthcare behaviors resulting from insurance coverage[13].

Moral hazard is known as one of the main causes of market failure [14] that has many adverse consequences, such as the impact on demand elasticity, reduction of welfare, inefficiency in using resources, reduction in technical and allocation efficiency, reduction of benefits of risk pooling, and price increase [11, 15]. Moral hazard, as a concern in the health insurance market, requires the application of appropriate policies and interventions to be controlled. In this regard, consumer moral hazard as the most obvious form of moral hazard [16] has been one of the topics of concern for policymakers and economic experts in recent years [8]. The aim of this study was to determine and analyze strategies used to control consumer moral hazards in health systems. The results of this study can be used for health insurance planning, health system financing, and health cost reduction.

## Methods

This study was written as part of a Ph.D thesis entitled “Developing a model to control consumer moral hazard in Iran’s health system” which was designed and performed based on its proposal and was approved by the local ethics committee of the Iran University of Medical Sciences (code: IR.IUMS.REC.1399.1103).

Research questions.

- What strategies or interventions are used to control consumers’ moral hazards?
- What is the approach of identifying strategies to control moral hazards?

### Databases and search strategies

For this systematic review, Web of Sciences, PubMed, Scopus, Embase, ProQuest (Dissertations database), and Iranian databases Magiran (the largest Iranian database in various scientific and specialized fields) and SID (open access database to Iranian Persian and English studies) were searched without time limitation, until the seventh of February 2021. In addition, to complete the search and ensure access to all related articles, the Google Scholar search engine was also searched. On July 21, 2022, the mentioned databases were researched to Identify new publications between February 2021and July 2022. During the new search five studies were added.

Search terms were used for the titles or abstracts of the records included “moral hazard”, “unnecessary use”, “unnecessary utilization”, “non-essential use”, “non-essential utilization”, “overutilization”, “health”, “health system”, “health insurance”, “health care”, “health service”, “health services”, “healthcare”, “medical care”, and “medical service”. In Web of Sciences, Scopus, and ProQuest due to their defined search strategy, in addition to the titles and abstracts, keywords were also searched. Search in any of the databases was performed using the defined search strategy of each database. The complete search strategy is shown in Additional file 1.

### Inclusion and exclusion criteria

Papers in English and Persian languages in the field of reducing and controlling consumer moral hazard in the health system, conducted in a quantitative, qualitative, and mixed methods design with theoretical and empirical approaches, that were of moderate and high quality based on Dixon Wood et al. ‘s checklist [17] were included. Abstracts, letters to the editors, conference, and seminar presentations were excluded.

### Methods of screening and selection criteria

**Fig. 1:**
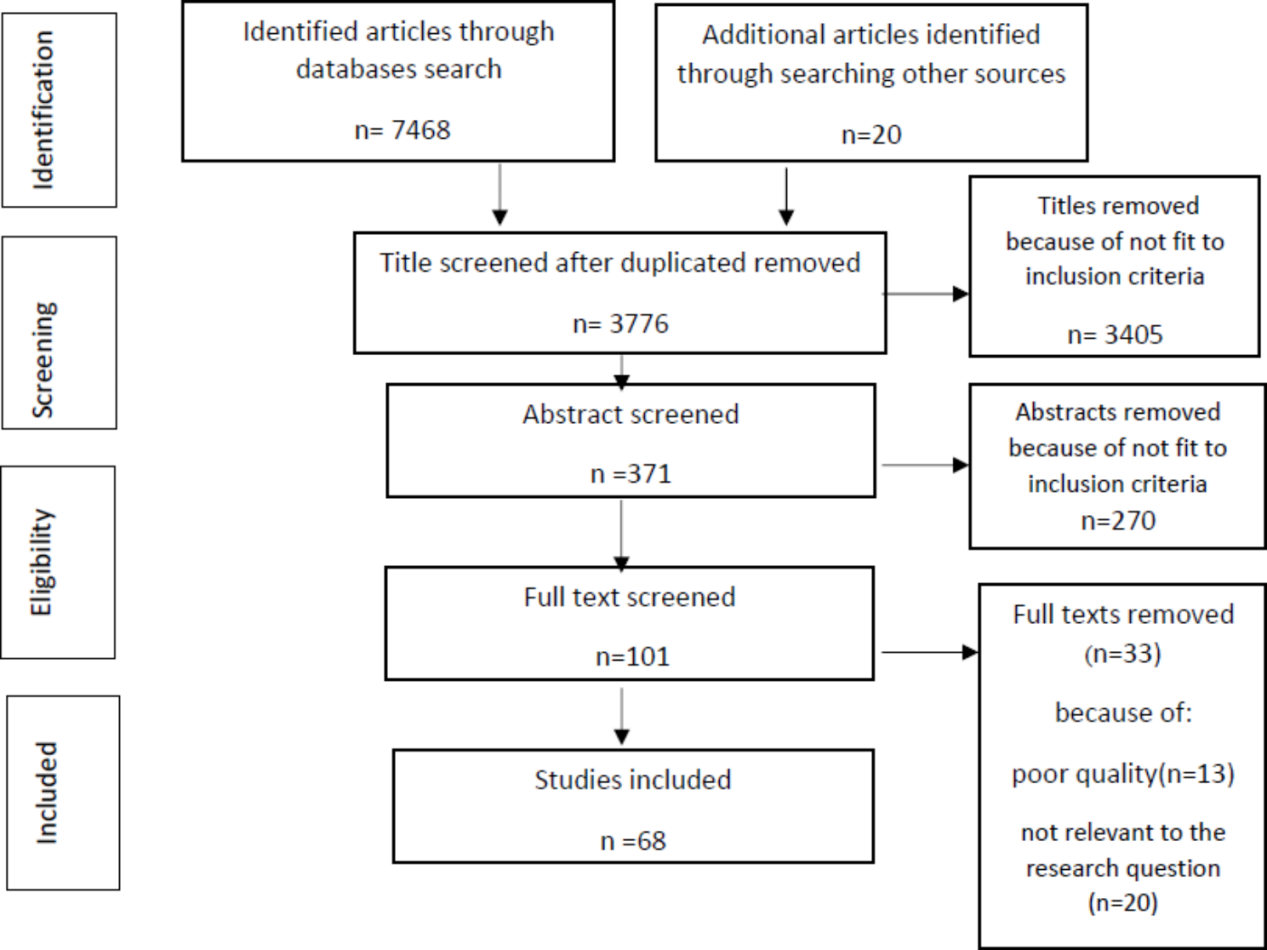
Literature selection and retrieval flow diagram.

All found articles were imported into Endnote software (version X9 (and duplicate articles were removed. Two researchers who were experts in the research topic and systematic review process independently screened the titles and abstracts of the articles (ZKR and MJ). In the final screening step, the full texts of the remaining articles were independently assessed by two researchers. Disagreements between the two researchers were resolved based on the opinion of a third researcher. Finally, the references of the retrieved articles were reviewed to find related articles that were not found in the first search. The screening process of retrieved papers is presented in Figure 1. Data extraction was conducted based on author’s name, title, year of publication, country, study design, strategies used to control consumer moral hazards, outcome variables, main results and quality assessment status. The main characteristics of the included study is shown in Additional file 2.

### Quality appraisal

In the first stage, the quality of the articles was assessed by two members of the research team who were familiar with the issue of moral hazard (ZKR and MJ), and the consensus was reached regarding the quality of the selected articles. The most important criteria for selecting high-quality articles were their relevance and role in the development of the study concept. Disagreements were resolved by a third author. The next step in the quality appraisal was based on Dixon-Woods et al. ‘s checklist [17], which included five questions regarding the clarity of the study objective(s), suitability of the study design to the objective(s), presentation of a clear report of the process of generating findings, use of sufficient data to support the interpretations, and use of appropriate analysis methods. The quality of the articles was determined based on 10 scores: 9–10 (high quality), 6–8 (moderate quality), and ≤ 5 (low quality). Articles with a score ≥ 6 were included in the study.

### Data analysis

A content analysis approach was used to summarize the findings of this qualitative systematic reviews. In this way, to achieve a general understanding, each article was read and re-read, and then each text was broken into small units called code; then, the codes were classified into categories based on their similarities and differences. After interpreting the categories, based on the purpose of the study, the main themes were identified. The process of coding and classifying the codes were done by two coders) ZKR and MJ).

## Results

In the search of databases and other sources, 7488 articles were retrieved, and after removing duplicate sources and applying inclusion and exclusion criteria, 68 eligible articles were selected (Fig. 1). General description of the selected studies is shown in Table 1. As Table 1 shows, out of 68 included papers, the majority of studies were conducted in a period from 2016 to 2021(n = 29), in a quantitative approach (n = 46). And, health service demand and utilization was the most common outcome investigated in these studies. (n = 38).

**Table 1:** General description of selected studies

Classification category	Sub category	Num	resources
Year of publication	1990–2000	1	[18]
	2001–2005	10	[19–28]
	2006–2010	11	[6, 29–38]
	2011–2015	17	[5, 9, 39–53]
	2016–2021	29	[10, 13, 15, 54–79]
Study design			
	Quantitative	46	[5,6,9, 13, 20, 22–25, 28, 32, 33, 35–38, 40–46, 48, 51–55, 57, 58, 61–67, 69, 71–76, 78]
	Qualitative	5	[26, 27, 39, 49, 70]
	Review	5	[10, 15, 47, 56, 60]
	Theatrical approach(model – based)	12	[18, 19, 21, 29–31, 34, 50, 59, 68, 77, 79]
Outcomes variable			
Demand & utilization		38	[5, 15, 20, 22, 23, 28, 30–33, 35, 36, 39–41, 43–48, 52, 54–56, 59, 61, 63–66, 69, 71–74, 77, 78]
Cost & optimality		26	[5, 15, 18, 19, 29–31, 34, 35, 37–40, 50, 51, 53, 57, 58, 60, 68, 71, 73–76, 79]
Health related behavior		2	[13, 46, 50, 77]
Opinion & acceptance		4	[24, 27, 49, 70]
Choice of plan		2	[42, 69]

The content analysis of 68 studies included in the study are presented in the form of two group, six themes, and 11 categories.

Since, based on the moral hazard theory, this phenomenon is defined as a change in consumer behavior because of insurance coverage, focusing on changing behavior and modifying it is the main goal of the controlling strategies. Therefore, the results of this study were summarized in the two groups: “Changing behavior at the time of receiving health services” (Table 2) and “Changing behavior before needing health services” (Table 3). The first group includes four themes: demand-side cost-sharing or consumer cost-sharing, health savings accounts, drug price regulation, and rationing for health services. The second group includes two themes: development of incentive insurance programs and community empowerment.

**Fig. 2:**
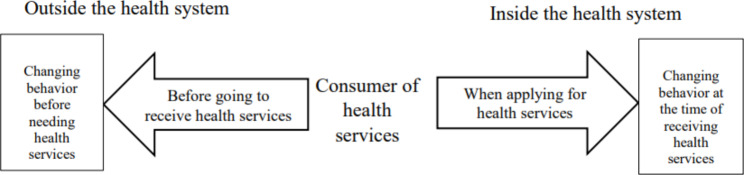
Relationship of strategies of “Changing behavior before needing health services” with strategies of “Changing behavior at the time of receiving health services”

The relationship between these two groups is drawn in the form of a diagram (Fig. 2.)

In reviewing the findings of the review studies, the results of four primary studies [18–21] were repeated in one review study [22] and the results of one primary study [23] were repeated in another review study [24]. Since in the qualitative analysis, the criteria for analyzing the findings are different from the quantitative results, repeated findings were not excluded in the qualitative analysis, but in the narrative report of the findings of quantitative studies, only the findings of the primary studies were presented.

### Strategies for changing behavior at the time of receiving health services

The themes (strategies) in this group, which are used when consumers refer to the health system and receive health services by increasing awareness of services costs and other indirect financial consequences (waiting), attempt to increase their responsibility to reduce the consumption of unnecessary health services. The themes of this group consider the changing consumptive behavior of health service consumers. This group includes four themes: demand-side cost-sharing or consumer cost-sharing, health savings accounts, drug price regulation, and rationing for health services.

#### Demand side cost sharing

Demand side cost sharing is a financial tool [25] and a kind of out-of-pocket payment [26] that is used to decrease the demand for health services or improve the utility of useful services [27], these strategies provide incentives to reduce unnecessary demands by paying part of the service cost by consumers [11]. Deductibles (The amount of health costs that a person must pay before the insurance begins to pay), copayment (paying a fixed amount of the cost of each health service unit), coinsurance (paying a percentage of the cost of each health service unit) [28], and capping (applying a cap on out-of-pocket payments or insurance claims) are different forms of cost sharing [15] that their design methods are diverse in insurance systems [29]. Cost sharing is often used in countries with social health insurance systems [30]. In the United States, it is also used in private insurance and Medicare and Medicaid systems [29].

The results of the study showed that *“demand side cost sharing“* or “consumer cost sharing” were the main strategies used for controlling consumer moral hazard as addressed by 56 studies [5, 6, 9, 10, 13, 15, 18–25, 30–71].

In our analysis, demand-side cost-sharing was divided into two categories: uniform cost-sharing and differential cost-sharing, which were classified based on the fixed or different rates of out-of-pocket payments in the form of deductible, copayment, and coinsurance.

#### Uniform cost-sharing

The uniform cost-sharing strategy are also known as traditional cost sharing methods includes strategies (codes) in which the rate of patients’ out-of-pocket payments (deductible, copayment, and coinsurance) is fixed or flat to use each unit of health services for different consumers.

#### Differential cost-sharing

In differential cost-sharing, deductibles, copayment, and coinsurance are adjusted based on criteria such as income level; health status; consumer choice, and the type, value, price, and elasticity of the health product or service. Differential cost - sharing have been proposed in response to the high sensitivity of low-income people [45, 49, 60, 64, 69] and inefficiency [64] of fixed and low cost - sharing strategies,

Strategies related to this category include income-based cost sharing, variable deductible plans (Shift deductible, variable deductible, different size of deductible. optional deductible, voluntary deductible (VD), high-deductible health plans (HDHPs), higher insurance deductibles), tier cost sharing (higher copay or coinsurance for services with higher price of the product or service(, value-based cost sharing (lower copay or coinsurance for services with higher costs benefits or lower elasticity), differential cost sharing based on disease status and Cost sharing with discount.

The studies included in this theme investigated consequences such as the utilization of health services, health costs, health related behavior, the choice of insurance plan, and the opinion and acceptance of consumers using quantitative, qualitative, review, and theoretical approaches (model-based), the results of quantitative studies are reported in narrative form as follows: modest to high reduction in health services and medications utilization [9, 20, 21, 23, 33, 38, 44, 45, 47, 49, 51, 55, 56, 64, 69, 71], reduction in health cost [21, 39, 41, 42, 44, 53, 65, 68], low or no significant effect on health services utilization [6, 34, 35, 40, 62, 63, 65], low or no significant effect on health costs [5], increasing cost contaminate incentive (CCI) [57], significant correlation with higher preventive behavior [13, 50] modest efficiency gain [18], increasing medication adherence [48], increasing demand for low price drug [61], optimal insurance [54].

The reported negative consequences are as follows: Decreasing the utilization of both necessary and unnecessary care [44, 55, 71], substitution effect from cares with cost-sharing to free care or with lower cost sharing [18, 70], and vulnerability of low-income groups [49, 60, 68].

Despite the focus of differential strategies on eliminating the shortcomings of uniform strategies, some studies indicate the ineffectiveness of these strategies in response to the problem of reduced consumption of non-essential services [44], delayed treatment, and medical debt in the vulnerable group [68]. Also, the low level of unhealthy behavior in members of HDHP may be due to the individual characteristics of the people who chose the plan not the impact of the plan [50].

### Health savings accounts (HSAs)

HSAs are considered as alternative tools for financing and dealing with future demographic challenges [72]. This financing theme under the title of health savings accounts [73, 74] or medical savings accounts [72, 75, 76], are currently used to decrease moral hazard and cost in four countries around the world [72, 75]. In this mechanism, owners of savings accounts should save a certain percentage of their income in these accounts for future health expenses. Funds of these accounts are used to pay for health expenses. Saving accounts increase people’s motivation to take responsibility by providing tax benefits and informed participation in health care decisions based on cost awareness and monitoring of physicians’ decisions [73].

In the United States and South Africa, they are used in combination with private insurance and is voluntarily. In Singapore and China, health saving accounts are governmental and compulsory which are implemented in combination with social health insurance and social risk-pooling system, respectively [72, 75]. This strategy in private health insurance and combination with high deductible health plans (HDHPs) is known as consumer directed health plans or consumer-driven health plans (CDHPs) [59].

In our study, health savings accounts were divided into two categories: voluntary health savings accounts (HSAs) and compulsory health savings accounts (HSAs), which were classified according to the mandatory and optional nature of the plan and the type of financing system in each country.

#### Voluntary health savings accounts

In this category, health savings accounts are optional, and in combination with private insurance and high deductible health plans (HDHPs)that is known as consumer directed health plans or consumer-driven health plans (CDHPs)[59]. In the United States and South Africa, this strategy are used [75].

#### Compulsory health savings accounts

Compulsory health savings accounts are governmental and compulsory which are implemented in combination with social health insurance and social risk-pooling system. Medisave (Medical savings accounts) (MSAs) in combination with social health insurance, Tongdao (MSA) in combination social risk-pooling (SRP) and Bankuai: (MSA) separately to finance outpatient services, are related to this category [75].

Findings related to health savings accounts were extracted from seven studies [55, 59, 72–76]. In studies related to this theme, the outcome such as health services utilization, health costs, and health promotion behaviors was assess using quantitative, review, and model-based theoretical approaches. The narrative reports of these outcomes in the quantitative studies are as follows: reduction in health services utilization and cost [75], useful for future savings [75] No or less effective in controlling healthcare costs [74, 76].

Some experts regard the usefulness of precautionary savings as positive point of these strategies [72]. In a study that assessed the effect of these strategies for prevention efforts and precautionary savings, it is stated that consumers do not take these two measures at the same time; in case of precautionary savings, preventive action is reduced, and vice versa [73]. Adverse selection, consequences of inflation, reduction of equity, and restraint of essential consumption are other negative consequences of this strategy [74].

### Drug price regulation

Drug pricing is an influential component of drug access and rational use of drugs. In addition to improving access, consumption management should be considered [77]. Drug price regulation is the third theme of this group, with three categories, uniform pricing, discriminatory pricing and two part pricing, which are based on drug pricing policies and the fix or different prices for each drug unit. As the findings of this theme were extracted from only one study, the categories related to this theme included one code that could not be combined and summarized further due to dissimilarity.

#### Uniform pricing

Uniform pricing refers to strategies in which a product is offered at the same price for all market segment regardless of the characteristics of each segments and its ability to pay [78]. It is one of the traditional methods of pricing in the pharmaceutical industry [79]. This pricing method, despite the ease of administration, is not able to satisfy all market segments. From the perspective of high-level customers, the suggested price may be low and indicate low desirability, whereas low-income customers may consider the price high and avoid buying it [80]. Results related to this category were extracted from a study [81].

#### Discriminatory pricing

Discriminatory pricing offers different prices for the same drug in different markets or groups [78]. Price discrimination is caused by the inability of developing countries to provide the medicines they need. Discriminatory pricing involves a segmented market that charges different prices based on each country’s ability to pay [77]. This category includes different types; however, in this study, only third degree price discrimination was introduced as an intervention to control moral hazard [81].

#### Two part pricing

Two-part pricing, another name for two-part tariffs, determines the price of medicine from the combination of uniform price and lump-sum payments [81] which has recently been proposed instead of uniform pricing for drugs [79].

Results related to the “drug pricing” theme were extracted from a study [81]. In this study, three types of pricing mechanisms, including uniform pricing, two-part tariffs and third degree price discrimination were compared in order to control the consumer’s moral hazard, the results showed two-part tariffs were considered a better strategy to address consumer moral hazard [81].

### Rationing of health services

Rationing of health services based on the waiting list (number) and waiting time (period) is one of the demand management strategies for non-emergency and elective health services [82]. Rationing of health services is the last theme that has one category named “rationing by waiting” which refers to strategies that control the consumer moral hazard by considering the cost of lost time. This strategy is one of the ways to reduce health costs that replaces user payments in countries without this system (national health system) to control costs and reduces unnecessary demand by imposing costs through the queue [30]. Findings related to the *“*Rationing of health services*”* were extracted from three studies [10, 30, 83]. These three studies investigated this mechanism using review and model-based theoretical approaches. The results of the theoretical analysis of these strategies regarding optimality [30] and well-being [83] outcomes were not associated with positive results.These strategies are not very popular and people tend to pay instead of waiting [83].

**Table 2:** Strategies for changing behavior at the time of receiving health services

Theme /source	Category	Code			Outcome variables		
Demand side cost sharing [5, 6, 9, 10, 13, 15, 18–20, 22–25, 27–33, 35–49, 51–57, 59–67, 70–73, 78]	Uniform cost sharing		- Fixed rate of deductible: traditional deductible, mandatory deductible, First euro deductible/ a first-dollar deductible, doughnut hole deductible - Fixed rate of co-payments or coinsurance/co-insurance: /copayment/ user charges / user-fee, lump sum co-payments, fixed payment, Mandatory co-payments - Fixed-rate insurance coverage limit /caps on insurance /stop loss. / payment ceiling limit on coverage, limit on out-of-pocket expenses		- Health services utilization - Health costs - Health related behavior - Choice of insurance plan - The opinion and acceptance of consumers		
	Differential cost sharing		- Income base cost sharing: income base deductible: income-related copayment /coinsurance/ different copayment according to the socioeconomic status - Varied deducible: Shift deductible, variable deductible, different size of deductible. optional deductible, voluntary deductible (VD), high-deductible health plans (HDHPs), higher insurance deductibles - Tier cost sharing: :multitier copayments / tiered copayment/ price-related co-payment tier, tier coinsurance - Value-based cost sharing: Value Based Insurance Design (VBID) / value-based cost sharing (lower coinsurance for services with higher costs benefits / value-based coinsurance target / variable co-insurance based on demand elasticity/ treatment-specific copayments disease-specific cost sharing/ differential cost sharing based on disease status - Cost sharing with discount: co-payment exemption, co-payment with rebate, / waiving copays				
Health savings accounts (HSAs) [26, 50, 54, 58, 60, 74, 75]	Voluntary Health Savings Accounts (HSAs)		- Health savings accounts (HSAs) / Health Reimbursement Accounts (HRAs) / medical savings accounts (MSAs) in combination with private insurance - Consumer-Directed Health Plans (CDHPS) /Consumer-Driven Health Plans (CDHPS) / Three-Tier Payment System(HDHPS Coupled With Personal Savings Account)	- Health services utilization - Health costs - Saving for future			
	Compulsory Health Savings Accounts (HSAs)		- Medisave (Medical savings accounts) (MSAs) in combination with social health insurance - Tongdao (MSA )in combination social risk-pooling (SRP)/ medical savings accounts (MSAs) in combination with social insurance pool (SIP)/ the three-tiered design (MSA-deductible-SIP) - Bankuai (MSA) separately to finance outpatient services				
Drug price regulation [21]	Uniform pricing discriminatory pricing		- Uniform pricing/uniform monopoly pricing - Third degree price discrimination / (different prices to different markets or groups) of consumers		- Drug utilization		
	Two PartPricing		- Two-part tariffs.) combines a uniform price with market-specific lump-sum payments				
Rationing for health services [10, 31, 34]	Rationing by waiting		- Waiting lists / queuing /expectancy queue/the cost of the lost time - Waiting time			- Optimality - Well-being	

### Strategies for changing behavior before needing health services

The themes in this group deal with the strategies that are applied outside the health system, before the need for healthcare services, and through the consciousness of health and positive financial incentives, increase healthy behavior or prevent unhealthy behavior. Changing individual behaviors to reduce high-risk behaviors and improve health-promoting behaviors is the approach of this group. This group includes two themes: development of incentive insurance programs and community empowerment.

### Development of incentive insurance programs

The themes of this group focus on the measures of insurance companies and purchasers. This theme refers to strategies aimed at reducing the risk of disease and the need for health services or unhealthy behaviors and consists of two categories: extending preventive care insurance and developing bonus-oriented insurance. Findings related to this theme were extracted from11 studies [10, 15, 25, 37, 43, 46, 58, 84–87].

#### Extending preventive care insurance

Expanding preventive care insurance refers to strategies that, by developing various types of preventive insurance, sensitize consumers to their health and reduce the need for more health services in the future by preventing the deterioration of their health status. Additionally, these strategies prevent the demand for specialized and expensive services by providing medium insurance plans [37, 84]. This category includes the following strategies (code): proposing insurance coverage for preventive care [37], separating insurance coverage for prevention and treatment [37], to encourage insureds to use more secondary preventive care [84] and improving perception of health status through secondary preventive care [84].

#### Developing bonus-oriented insurance

These strategies reduce unnecessary consumption by providing incentives to avoid inefficient service. In these strategies, insurance attempts to control consumers’ moral hazard by applying positive financial incentives in the form of premium discounts [10], or more coverage [86] in the following year’s contract, in the case of less service consumption or applying preventive effort Bonuses for non-consumption or limited consumption are often used in risk adjustment schemes [58].

Health service utilization, health costs, risk-reducing behaviors, and choice of expensive health services were among the variables investigated in studies of this them in quantitative, qualitative, and theoretical approaches. The results of these studies can be summarized as follows: more feasibility to incentivize consumers to purchase more secondary preventive care [84] higher reduction in moral hazard in the copayment with a premium reduction frame than copayment reduction frame [46] moral hazard reduction in voluntary deductible is expected to be larger in a system with risk-rated premiums than in a system with community-rated premiums [85].

### Community empowerment

Community empowerment is the second and last theme in this group with one category called Community education.The theme of ”community empowerment”, mentioned by only one study [33].

#### Community education

This category refers to the development of health-promoting behaviors through community education and increasing people’s awareness of the function of insurance and the consequences of the unnecessary use of health services by using the capacity of civil society [33]. Despite the fact that only one study had dealt with this issue marginally, due to the importance of the subject, the research team decided to set this code as an independent theme.

The sources from which each code is extracted are provided in Additional file 3.

### Risk of bias consideration

The risk of bias assessment in this study consisted of the following: To reduce publication bias, unpublished papers were searched in the Dissertations database of ProQuest for grey literature, but no related papers were found. In this regard, there is a possibility of language bias due to the limitation of non-English articles in publishing or indexing the results and the focus of this study on Persian and English articles, which is mentioned as a limitation in the limitations section.

**Table 3:** Strategies for changing behavior before needing health services

Theme/ source	Category	Code	Outcome variables
Development of incentive insurance programs [10, 15, 27, 30, 39, 42, 59, 69, 76, 77, 79]	Extending preventive care insurance	- Proposing insurance coverage for preventive care - Separating insurance coverage for prevention and treatment - To encourage insureds to use more secondary preventive care - Improving perception of health status through secondary preventive care	- Health services utilization - Health costs - Risk-reducing behaviors - Choice of expensive health services health
	Development of bonus oriented insurance	- Adjusted premiums according level of preventive effort - Premium reduction, bonus payments/ rebate insurance /to encourage non-use or limited use in return for next premium reduction, risk adjustment .no-claims bonus, risk rating premium - No-claim Bonus and Coverage Upper Bound	
Community empowerment [23]	Community education	- Health promotion education - Civic education about the consequences of unnecessary use of health services	- Not evaluated

## Discussion

The aim of this study was to determine and analyze strategies used to control consumer moral hazards in health systems. A wide range of goals, approaches, and various research designs have been investigated and reported.

Controlling strategies for consumer moral hazard are known as demand-side strategies whose goal are to motivate consumers to reduce unnecessary demand or consumption. In this study, the strategies to control the consumer moral hazard were divided into two groups.The first group aims to control consumer consumptive behavior when receiving health services. The second group focuses on reducing the need for health services by controlling health-related behaviors before needing health services. This classification of controlling strategies was taken from the approach of dividing moral hazards into ex-post and ex-ante moral hazard. Ex-post moral hazard means an increase in demand for health services due to price reduction, which indicates consumer price sensitivity [85]. Ex-ante moral hazard refers to a reduction in preventive behaviors and an increase in risky behaviors due to insurance coverage [88, 89].

The results of this study show a greater frequency of studies related to the strategies of the first group and control of consumptive behavior, In contrast, strategies used for changing health-related behavior are limited which indicates that researchers pay more attention to ex-post moral hazard. Ex-post moral hazard has been widely studied, but evidence of ex-ante moral hazard is very limited [88, 90]. The reason for less attention paid to the ex-ante moral hazard modeled by Ehrlich and Becker in 1972 may be criticized as follows: cost is not the only consequence of illness that, if paid by someone else, makes people indifferent to their healthcare [90, 91].

Another noteworthy point of this study’s findings is the financial nature of most strategies, including demand-side cost sharing as one of the most effective methods, health savings accounts, and drug pricing. Imposing a cost through the waiting list can also be considered a financial tool. Access restrictions caused by the negative financial incentives is one of the adverse effects of these strategies. A recent study on cost sharing showed a significant relationship between cost sharing and adult mortality in poor countries. The authors believe that this issue should be considered when analyzing the social welfare consequences of cost-sharing [92]. Also, during the analysis, Michaela et al. stated that medical savings accounts cause inequality and provide little financial protection [93].

Although bonus insurance is a positive incentive financial tool because the individual receives a reward in the form of a premium discount or more coverage in exchange for reduced or non-consumption, the results of a qualitative study in insured individuals proved these strategies to be less optimistic and justified compared to demand-side cost sharing strategies [25]. In addition, the community empowerment strategy, despite being a non-financial tool, needs further investigation in future studies due to the limitations of effective studies.

The outcomes analyzed in the included studies are other points of debate in this review. The majority of the outcomes analyzed included demand and utilization of health services [5, 15, 18–24, 30, 33, 37–40, 43–45, 47–51, 55, 56, 58, 60, 62–65, 68–71, 75, 84, 86] and health services costs and expenditures, optimality and efficiency of strategies [5, 15, 30–32, 36, 37, 39, 41–44, 53, 54, 57, 59, 68, 70, 73–76, 83, 85, 87, 94]. Limited studies have addressed other important aspects such as access to low-income people [49, 60, 68], reduction in the consumption of both essential (such as preventive and diagnostic services) and non-essential services [24, 44, 58], and people’s attitudes and acceptance [25, 34, 52, 67].

The impact of strategies on outcomes such as the utilization of health services and health costs has been different, which seems normal due to the different implementation and management methods and whether the programs are mandatory or optional. However, in this context, the point to consider is to pay attention to the negative consequences, including the higher sensitivity of low-income groups and the shifting financial burden to insureds and an increase in total costs of health costs due to the substitution effect especially in cost-sharing methods. Thesee consequences challenge the achievement of equity in the access and efficiency of the health system. Since moral hazard is one of the factors of the inefficiency of the health system [95] therefore, in its control, improving efficiency should be the most important goal.

In this regard, the results of this research showed that controlling strategies need further investigation in future study. Due to the focus of most studies on the controlling strategies of consumptive behavior at the point of receiving the service, therefore, the suggestions are as follows. Reviewing existing strategies, especially strategies with negative financial incentives to minimize adverse consequences, paying more attention to current strategies from the perspective of preventing the need for health services and the introduction of new strategies with preferably non-financial approaches that do not limit access. Obviously, in the design of new interventions and revision of existing interventions, important consequences such as access, financial protection, equality, and quality of services provided along with service utilization and service costs should be taken into consideration.

On the other hand, considering that each of these strategies is used in different health systems with different financing mechanisms, so managers in each health system need to adjust strategy to the characteristics of their health system. In addition, considering the nature of behavioral change of strategies, knowing the characteristics of consumers, the pattern and culture of health service consumption and their health-related behaviors is the first step to choosing the most appropriate strategy and adapting it to each Society’s conditions.

## Limitations

This study has several limitations. The first, is the restriction of studies in Persian and English languages. No clear boundary between the consumer and provider moral hazard in some articles is another limitation. The researchers separated these two issues by studying the full text of the articles, focusing on the type and setting of service delivery and the role of physicians in providing services. The last limitation was the methodological diversity and heterogeneity of the quantitative studies, which did not allow for quantitative analysis and reporting the effectiveness of the strategies.

## Conclusion

Strategies to control consumer moral hazards focus on changing consumer consumptive and health-related behaviors, which are designed according to the structure of health and financing systems. Since “changing consumptive behavior” strategies are the most commonly used strategies; therefore, it is necessary to strengthen strategies to control health-related behaviors and develop new strategies in future studies. In addition, in the application of existing strategies, the adaptation to the structure of the health and financing system, and the pattern of consumption of health services in society should be considered.

## Electronic supplementary material

Below is the link to the electronic supplementary material.

Supplementary Material 1

Supplementary Material 2

Supplementary Material 3

## Data Availability

All data are within the manuscript and additional files.

## Abbreviations

OECD: Organization for Economic Cooperation and Development.
GDP: Gross Domestic Product.
VBID: Value Based Insurance Design.
HDHPs: High Deductible Health Plans.
CDHPs: Consumer-Directed Health Plans or Consumer-Driven Health Plans.
MSAs: Medical Savings Accounts.
HASs: Health Savings Accounts.
HRA: Health Reimbursement Accounts.
SRP: social risk-pooling.
SIP: social insurance pool.
