# Supplementary material for: A systematic review of strategies used for controlling consumer moral hazard in health systems
Source: BMC Health Serv Res. 2022 Oct 18;22:1260. doi: 10.1186/s12913-022-08613-y (PMC9580205; doi:10.1186/s12913-022-08613-y)
Supplement: Supplementary file 1 — Supplementary Material 1 [file 12913_2022_8613_MOESM1_ESM.docx]

**Additional file 1. Complete Search Strategy**

| **Databases** | **Search strategy** | **Result** |
| --- | --- | --- |
| **PubMed** | (“moral hazard”[tiab] OR “moral hazards”[tiab] OR “principal agency problem”[tiab] OR “principal agent dilemma”[tiab] OR “principal agent problem”[tiab] OR “unnecessary use”[tiab] OR **“**unnecessary utilization”[tiab] OR **“**non-essential use”[tiab] OR **“**non essential utilization”[tiab] OR overutilization[tiab] OR overutilizations[tiab] OR overutilization[tiab] OR overutilisations[tiab] OR **“**over-utilization”[tiab] OR “over-utilizations”[tiab] OR **“**over-utilisation”[tiab] OR “over-utilisations”[tiab]) AND (“Delivery of Healthcare”[tiab] OR “Healthcare Deliveries”[tiab] OR “Healthcare Delivery”[tiab] OR (Deliveries[tiab] AND Healthcare[tiab]) OR (Delivery[tiab] AND Healthcare[tiab]) OR “Health Care Delivery”[tiab] OR (Delivery[tiab] AND “Health Care”[tiab]) OR “Health Care”[tiab] OR (Care[tiab] AND Health[tiab]) OR Healthcare[tiab] OR “Health Care Systems”[tiab] OR “Health Care System”[tiab] OR (System[tiab] AND “Health Care”[tiab]) OR (Systems[tiab] AND “Health Care”[tiab]) OR “Healthcare Systems”[tiab] OR “Healthcare System”[tiab] OR (System[tiab] AND Healthcare[tiab]) OR (Systems[tiab] AND Healthcare[tiab]) OR “Community-Based Distribution”[tiab] OR “Community Based Distribution”[tiab] OR “Community-Based Distributions”[tiab] OR (Distribution[tiab] AND “Community-Based”[tiab]) OR (Distributions[tiab] AND “Community-Based”[tiab]) OR “health system”[tiab] OR “long stay care”[tiab] OR “long term care”[tiab] OR “health insurance”[tiab] OR “health service”[tiab] OR “health services"[tiab] OR “medical care“[tiab] OR “medical service”[tiab] OR “medical services”[tiab] OR drug[tiab] OR medication[tiab] OR outpatient[tiab] OR "physician visit"[tiab] OR "outpatient visit"[tiab] OR inpatient[tiab] OR hospitalization[tiab] OR hospitalization[tiab] OR “hospital admission”[tiab] OR “hospital care”[tiab]) | 1128 |
| **Embase** | (“moral hazard”:ti,ab OR “moral hazards”:ti,ab OR “principal agency problem”:ti,ab OR “principal agent dilemma”:ti,ab OR “principal agent problem”:ti,ab OR “unnecessary use”:ti,ab OR **“**unnecessary utilization”:ti,ab OR **“**non-essential use”:ti,ab OR **“**non essential utilization”:ti,ab OR overutilization:ti,ab OR overutilizations:ti,ab OR overutilization:ti,ab OR overutilisations:ti,ab OR **“**over-utilization”:ti,ab OR “over-utilizations”:ti,ab OR **“**over-utilisation”:ti,ab OR “over-utilisations”:ti,ab) AND (“Delivery of Healthcare”:ti,ab OR “Healthcare Deliveries”:ti,ab OR “Healthcare Delivery”:ti,ab OR (Deliveries:ti,ab AND Healthcare:ti,ab) OR (Delivery:ti,ab AND Healthcare:ti,ab) OR “Health Care Delivery”:ti,ab OR (Delivery:ti,ab AND “Health Care”:ti,ab) OR “Health Care”:ti,ab OR (Care:ti,ab AND Health:ti,ab) OR Healthcare:ti,ab OR “Health Care Systems”:ti,ab OR “Health Care System”:ti,ab OR (System:ti,ab AND “Health Care”:ti,ab) OR (Systems:ti,ab AND “Health Care”:ti,ab) OR “Healthcare Systems”:ti,ab OR “Healthcare System”:ti,ab OR (System:ti,ab AND Healthcare:ti,ab) OR (Systems:ti,ab AND Healthcare:ti,ab) OR “Community-Based Distribution”:ti,ab OR “Community Based Distribution”:ti,ab OR “Community-Based Distributions”:ti,ab OR (Distribution:ti,ab AND “Community-Based”:ti,ab) OR (Distributions:ti,ab AND “Community-Based”:ti,ab) OR “health system”:ti,ab OR “long stay care”:ti,ab OR “long term care”:ti,ab OR “health insurance”:ti,ab OR “health service”:ti,ab OR “health services":ti,ab OR “medical care“:ti,ab OR “medical service”:ti,ab OR “medical services”:ti,ab OR drug:ti,ab OR medication:ti,ab OR outpatient:ti,ab OR "physician visit":ti,ab OR "outpatient visit":ti,ab OR inpatient:ti,ab OR hospitalization:ti,ab OR hospitalization:ti,ab OR “hospital admission”:ti,ab OR “hospital care”:ti,ab) | 1752 |
| **Scopus** | (TITLE-ABS-KEY(“moral hazard”) OR TITLE-ABS-KEY(“moral hazards”) OR TITLE-ABS-KEY(“principal agency problem”) OR TITLE-ABS-KEY(“principal agent dilemma”) OR TITLE-ABS-KEY(“principal agent problem”) OR TITLE-ABS-KEY(“unnecessary use”) OR TITLE-ABS-KEY(**“**unnecessary utilization”) OR TITLE-ABS-KEY(**“**non-essential use”) OR TITLE-ABS-KEY(**“**non essential utilization”) OR TITLE-ABS-KEY(overutilization) OR TITLE-ABS-KEY(overutilizations) OR TITLE-ABS-KEY(overutilization) OR TITLE-ABS-KEY(overutilisations) OR TITLE-ABS-KEY(**“**over-utilization”) OR TITLE-ABS-KEY(“over-utilizations”) OR TITLE-ABS-KEY(**“**over-utilisation”) OR TITLE-ABS-KEY(“over-utilisations”)) AND (TITLE-ABS-KEY(“Delivery of Healthcare”) OR TITLE-ABS-KEY(“Healthcare Deliveries”) OR TITLE-ABS-KEY(“Healthcare Delivery”) OR (TITLE-ABS-KEY(Deliveries) AND TITLE-ABS-KEY(Healthcare)) OR (TITLE-ABS-KEY(Delivery) AND TITLE-ABS-KEY(Healthcare)) OR TITLE-ABS-KEY(“Health Care Delivery”) OR (TITLE-ABS-KEY(Delivery) AND TITLE-ABS-KEY(“Health Care”)) OR TITLE-ABS-KEY(“Health Care”) OR (TITLE-ABS-KEY(Care) AND TITLE-ABS-KEY(Health)) OR TITLE-ABS-KEY(Healthcare) OR TITLE-ABS-KEY(“Health Care Systems”) OR TITLE-ABS-KEY(“Health Care System”) OR (TITLE-ABS-KEY(System) AND TITLE-ABS-KEY(“Health Care”)) OR (TITLE-ABS-KEY(Systems) AND TITLE-ABS-KEY(“Health Care”)) OR TITLE-ABS-KEY(“Healthcare Systems”) OR TITLE-ABS-KEY(“Healthcare System”) OR (TITLE-ABS-KEY(System) AND TITLE-ABS-KEY(Healthcare)) OR (TITLE-ABS-KEY(Systems) AND TITLE-ABS-KEY(Healthcare)) OR TITLE-ABS-KEY(“Community-Based Distribution”) OR TITLE-ABS-KEY(“Community Based Distribution”) OR TITLE-ABS-KEY(“Community-Based Distributions”) OR (TITLE-ABS-KEY(Distribution) AND TITLE-ABS-KEY(“Community-Based”)) OR (TITLE-ABS-KEY(Distributions) AND TITLE-ABS-KEY(“Community-Based”)) OR TITLE-ABS-KEY(“health system”) OR TITLE-ABS-KEY(“long stay care”) OR TITLE-ABS-KEY(“long term care”) OR TITLE-ABS-KEY(“health insurance”) OR TITLE-ABS-KEY(“health service”) OR TITLE-ABS-KEY(“health services") OR TITLE-ABS-KEY(“medical care“) OR TITLE-ABS-KEY(“medical service”) OR TITLE-ABS-KEY(“medical services”) OR TITLE-ABS-KEY(drug) OR TITLE-ABS-KEY(medication) OR TITLE-ABS-KEY(outpatient) OR TITLE-ABS-KEY("physician visit") OR TITLE-ABS-KEY("outpatient visit") OR TITLE-ABS-KEY(inpatient) OR TITLE-ABS-KEY(hospitalization) OR TITLE-ABS-KEY(hospitalization) OR TITLE-ABS-KEY(“hospital admission”) OR TITLE-ABS-KEY(“hospital care”)) | 2282 |
| **Web of Science** | (TS=(“moral hazard”) OR TS=(“moral hazards”) OR TS=(“principal agency problem”) OR TS=(“principal agent dilemma”) OR TS=(“principal agent problem”) OR TS=(“unnecessary use”) OR TS=(**“**unnecessary utilization”) OR TS=(**“**non-essential use”) OR TS=(**“**non essential utilization”) OR TS=(overutilization) OR TS=(overutilizations) OR TS=(overutilization) OR TS=(overutilisations) OR TS=(**“**over-utilization”) OR TS=(“over-utilizations”) OR TS=(**“**over-utilisation”) OR TS=(“over-utilisations”)) AND (TS=(“Delivery of Healthcare”) OR TS=(“Healthcare Deliveries”) OR TS=(“Healthcare Delivery”) OR (TS=(Deliveries) AND TS=(Healthcare)) OR (TS=(Delivery) AND TS=(Healthcare)) OR TS=(“Health Care Delivery”) OR (TS=(Delivery) AND TS=(“Health Care”)) OR TS=(“Health Care”) OR (TS=(Care) AND TS=(Health)) OR TS=(Healthcare) OR TS=(“Health Care Systems”) OR TS=(“Health Care System”) OR (TS=(System) AND TS=(“Health Care”)) OR (TS=(Systems) AND TS=(“Health Care”)) OR TS=(“Healthcare Systems”) OR TS=(“Healthcare System”) OR (TS=(System) AND TS=(Healthcare)) OR (TS=(Systems) AND TS=(Healthcare)) OR TS=(“Community-Based Distribution”) OR TS=(“Community Based Distribution”) OR TS=(“Community-Based Distributions”) OR (TS=(Distribution) AND TS=(“Community-Based”)) OR (TS=(Distributions) AND TS=(“Community-Based”)) OR TS=(“health system”) OR TS=(“long stay care”) OR TS=(“long term care”) OR TS=(“health insurance”) OR TS=(“health service”) OR TS=(“health services") OR TS=(“medical care“) OR TS=(“medical service”) OR TS=(“medical services”) OR TS=(drug) OR TS=(medication) OR TS=(outpatient) OR TS=("physician visit") OR TS=("outpatient visit") OR TS=(inpatient) OR TS=(hospitalization) OR TS=(hospitalization) OR TS=(“hospital admission”) OR TS=(“hospital care”)) | 1644 |
| **ProQuest** | TI,AB,SU(“moral hazard” OR “moral hazards” OR “principal agency problem” OR “principal agent dilemma” OR “principal agent problem” OR “unnecessary use” OR **“**unnecessary utilization” OR **“**non-essential use” OR **“**non essential utilization” OR overutilization OR overutilizations OR overutilization OR overutilisations OR **“**over-utilization” OR “over-utilizations” OR **“**over-utilisation” OR “over-utilisations”) AND TI,AB,SU(“Delivery of Healthcare” OR “Healthcare Deliveries” OR “Healthcare Delivery” OR (Deliveries AND Healthcare) OR (Delivery AND Healthcare) OR “Health Care Delivery” OR (Delivery AND “Health Care”) OR “Health Care” OR (Care AND Health) OR Healthcare OR “Health Care Systems” OR “Health Care System” OR (System AND “Health Care”) OR (Systems AND “Health Care”) OR “Healthcare Systems” OR “Healthcare System” OR (System AND Healthcare) OR (Systems AND Healthcare) OR “Community-Based Distribution” OR “Community Based Distribution” OR “Community-Based Distributions” OR (Distribution AND “Community-Based”) OR (Distributions AND “Community-Based”) OR “health system” OR “long stay care” OR “long term care” OR “health insurance” OR “health service” OR “health services" OR “medical care“ OR “medical service” OR “medical services” OR drug OR medication OR outpatient OR "physician visit" OR "outpatient visit" OR inpatient OR hospitalization OR hospitalization OR “hospital admission” OR “hospital care”) | 341 |
| **google scholar engine** | | **300** |
| **Iranian databases: Magiran and SID** | | **21** |
| **All record** | | **7468** |
