# Supplementary material for: A systematic review of strategies used for controlling consumer moral hazard in health systems
Source: BMC Health Serv Res. 2022 Oct 18;22:1260. doi: 10.1186/s12913-022-08613-y (PMC9580205; doi:10.1186/s12913-022-08613-y)
Supplement: Supplementary file 2 — Supplementary Material 2 [file 12913_2022_8613_MOESM2_ESM.docx]

| **source** | **Author/**  Additional file 2: The main characteristics of the included studies  **year /** | **Country/ language** | **Title** | **Design& Analyze** | **Control strategy** | **Outcome variable** | **Main results** | **Quality assessment** |
| --- | --- | --- | --- | --- | --- | --- | --- | --- |
| 1. [54] | Abdus S. 2020 | United States of America/ English | The role of plan choice in health care utilization of high-deductible plan enrollees. | quantitative/  statistic | high‐deductible health plan (HDHPs), consumer‐directed health plans (CDHPs), low‐deductible health plans (LDHPs), no‐deductible health plans (NDHPs). | health care utilization:  ambulatory visit, specialist visit, preventive services | lower levels of utilization in HDHP enrollees compared with those of the NDHP enrollees for any ambulatory visit, any specialist visit, and most preventive services | High quality |
| 1. [76] | Antonini m.etal  2021 | Chile/ English | Can risk rating increase the ability of voluntary deductibles  to reduce moral hazard? | quantitative/  statistic (empirical simulation) | voluntary deductibles& risk-rated premiums and community-rated premiums | moral hazard reduction(percentage of the corrected mean actual spending) | the absolute moral hazard reduction from a voluntary deductible is indeed expected to be larger in a system with risk-rated  premiums than in a system with community-rated premiums | High quality |
| 1. [55] | Alessie RJM, et al 2020 | Netherlands /English | Moral hazard and selection for voluntary deductibles. | quantitative/  statistic | voluntary deductible  with premium reduction/ rebate | moral hazard (GP visits, a medical specialist visits, number of days spent in a hospital, number of visits to mental health care | Reduction in moral hazard, especially in the number of visit a doctor and fewer days spending in the hospital | High quality |
| 1. [56] | Agarwal R, et al 2017 | United States of America/ English | High Deductible Health Plans  Reduce Health Care Cost And  Utilization,Including Use of Needed Preventive Services | systematic review | high-deductible health plans (HDHPs | health care utilization and health care costs | significant reduction in office visits, preventive care and medication adherence (reduction in both appropriate & inappropriate care) and  lower health care costs | High quality |
| 1. [39] | Bakx P et al 2015 | Germany, Belgium, Switzerland and the Netherlands | Demand-side strategies to deal with moral hazard in public insurance for long-term care. | qualitative study (comparative) | Cost sharing:  Copayments& deductibles  Managed competition: Financial risk and risk adjustment | Effect on access  Effect on efficiency | Cost sharing effect on efficiency: positive  Cost sharing effect on access: limited or negative Managed competition effect on access: limited or negative, Managed competition effect on efficiency: limited | Moderate quality |
| 1. [10] | Barati M et al 2018 | Iran /English | Moral hazards in providing health services: A review of studies. | Review | Deductible:  Fixed payment  Payment, Ceiling, encourage not to use or use to a limited extent  The first payment or the initial deductible:  Expectancy queue:  Health savings accounts: | - | policies need to be taken to prevent the worst misuseof resources by avoiding unnecessary and costly  expenditures and thus prevent higher costs and  inflation in the health sector. | High quality |
| 1. [19] | Bardey D & Lesur R. 2005 | France/ English | Optimal health insurance contract: Is a deductible useful? | Theoretical approach based on the assumptions of a model | Deductible | Optimal health insurance contract | for small diseases, may be optimal. for strong disease, full coverage is optimal. deductibles must be contingent at the severity of illness. | Moderate  quality |
| 1. [40] | Beeuwkes Buntin M, et al 2011 | United States of America/ English | Healthcare spending and preventive care in high‐deductible and consumer‐directed health plans | quantitative/  statistic | high deductible  health plans (HDHPs) & consumer directed  health plans (CDHPs) | Healthcare spending and use of recommended  preventive care | Reduction of healthcare  Spending and use of  preventive care in the first year. | Moderate  quality |
| 1. [77] | Benjiang M, et al. 2021 | China / English | Optimal insurance contract design with “No-claim Bonus and Coverage Upper Bound” under moral hazard. | theoretical approach based on model formulation | No-claim Bonus and Coverage Upper Bound | risk-reducing effort and utility | the combined incentive tool can restrain the moral hazard of the insured | Moderate  quality |
| 1. [57] | Cattel D, et al. 2017 | Netherlands/ English | method to simulate incentives for cost containment under various cost sharing designs: An application to a first-euro deductible and a doughnut hole. | developing a simulation  model / Statistic | different deductible  modalities: first-euro deductible and doughnut hole deductible | cost containment incentives  (CCI | CCI increases with the  deductible amount. different deductible modalities leads different CCIs | Moderate  quality |
| 1. [58] | Chen T. 2021, | China/ English | Can health savings account reduce health spending? Evidence from China | quantitative/  statistic | health savings accounts (HSAs) | containing medical expenses  reducing moral  hazard. | negative results for the effectiveness of HSAs in containing medical expenses and reducing moral hazard. Larger positive effect of HSAs on medical expenses for healthier group | Moderate  quality |
| 1. [18] | Chernew ME, et al 2000 | United States of America/ English | Optimal health insurance: The case of observable, severe illness. | Theoretical approach based on assumptions of a model (simulation) | optimal cost sharing provisions /Treatment-specific copayments | optimal insurance contracts for  reduction in moral hazard | reduction in moral hazard | Moderate  quality |
| 1. [41] | Choi Y, et al. 2015 | Korean/ English | The effect of cost-sharing in private health insurance on the utilization of health care services between private insurance purchasers and non-purchasers: A study of the Korean health panel survey (2008-2012). | quantitative/  statistic | Introduction Cost sharing in Privet health insurance (PHI | the number of outpatient visits, the number of inpatient visits, length of stay in hospital/ | the utilization of outpatient visits by purchasers decreased more than nonpurchases.  utilization of inpatient services was not statistically significant. | High  quality |
| 1. [20] | Cockx & Brasseur C. 2003 | Belgium/ English | The demand for physician services: Evidence from a natural experiment | quantitative/  statistic | To increase copayment rates of  three types of physician services | (GPs) visits, home visits, specialist visits add efficiency | small price elasticity of a uniform copayment, large substitution effects especially for women, modest efficiency gain | Moderate  quality |
| 1. [13] | Courbage C & Nicolas CJJoR 2021; | Swiss/  English | On the association between insurance deductibles and prevention behaviour: Evidence from the Swiss health system. | quantitative/  statistic | higher insurance  deductibles | Prevention Behaviour (good diet, exercising and limiting/abstaining from smoking and alcohol consumption) | except for alcohol  consumption, higher insurance deductibles were significantly associated with higher behavioural  prevention | High  quality |
| 1. [42] | Drevs F & Tscheulin.d k. 2013 | Germany/  English | The effect of framing on the choice of co-payment policies, reducing moral hazard and post-choice-evaluation. | quantitative/  statistic | choice of co-payment policies:  - co-payment with a rebate frame  -co-payment with a premium reduction frame | ex-post moral | reducing ex-post moral hazard is higher in the copayment with a premium reduction frame. | High  quality |
| 1. [43] | Ebrahimnia M, et al  2014 | Iran/ Persian | Impact of deductibles on insured moral hazard in the armed forces health services: A case study in Tehran. | quantitative/  statistic | coinsurance | Outpatient services  Inpatient services and medication | a negative relationship between moral hazard and coinsurance. Also increasing in income and age lead to increasing in moral hazard (relationship between marital status and moral hazard | Moderate  quality |
| 1. [30] | Ellis RP & Manning WG 2007 | United States of America/ English | Optimal health insurance for prevention and treatment | Theoretical approach based on assumptions of a model | Offer some insurance coverage for preventive care | Optimal health insurance | Proposing a more generous insurance coverage for prevention and treatment intervention | Moderate  quality |
| 1. [75] | Fan M et al 2016 | China  English | Discounting of medical  savings accounts | quantitative/  statistic | reduced MSA funds | health-care expenditures | less effective in controlling health-care costs. | high  quality |
| 1. [21] | Felder S  2004 | Germany/ English | Drug price regulation under consumer moral hazard. Two-part tariffs, uniform price or third-degree price discrimination? | theorical approach | Two-part tariffs, uniform price or third-degree price discrimination | Consumer moral hazard in drug consumption | The two-part tariff is better to address moral hazard. | Moderate  quality |
| 1. [31] | Felder S  2008 | Germany/ English | To wait or to pay for medical treatment? Restraining ex-post moral hazard in health insurance. | Theoretical approach based on assumptions of a model | queuing as a rationing device  waiting time and coinsurance | Optimal insurance contracts | an optimized design does not generally use a positive waiting time for medical treatment | Moderate  quality |
| 1. [59] | Fels M.. Health. 2020 | Germany/ English | reducing access: The case against cost-sharing in insurance | Theoretical approach based on assumptions of a model | Cost sharing and  bonus payments/ rebates insurance | access to efficient care | cost-sharing never to be an optimal strategy because of its side effect but bonuses, has no such side effects. | Moderate  quality |
| 1. [60] | Ferguson W, et al. 2020 | United States of America/ English | Potential savings from consumer-driven health plans. | Review | Consumer-Driven Health Plans/  Consumer engagement/  three-tier payment system | financial savings & transparency of healthcare cost. Moral hazard | improving healthcare price transparency and decreasing healthcare expenditures. | Moderate  quality |
| 1. [32] | Fiorio CV& Siciliani L. 2010. | Italy/ English | Co-payments and the demand for pharmaceuticals Evidence from Italy | quantitative/  statistic | To Increase copayment | per capita number of prescriptions  per capita public pharmaceutical expenditure | increasing in the co-payment reduces the per capita number of  prescriptions and per capita public pharmaceutical expenditure | high  quality |
| 1. [44] | Frank MB, et al. 2012 | United States of America/ English | The effect of a large regional health plan's value-based insurance design program on statin use. | quantitative/  statistic) | Value-based Insurance Design  Copayments on VBID brand statins | medication adherence( medication possession) | Medication adherence increased in the treatment group | High  quality |
| 1. [33] | Gerfin M & Schellhorn M. 2006 | Switzerland/English | Nonparametric bounds on the effect of deductibles in health care insurance on doctor visits - swiss evidence. | quantitative/  statistic | Different size of deductibles | the probability of going to the doctor | correlation between the degree of deductibles and health care health care utilization (reduction of moral hazard effects) | Moderate  quality |
| 1. [34] | Gravelle H, Siciliani L.. 2008 | United Kingdom / English | Optimal quality, waits and charges in health insurance | Theoretical approach based on assumptions of a model | waiting time | Optimal quality | no welfare gain from a positive waiting time. | Moderate  quality |
| 1. [61] | Hafner P& Mahlich JC. 2015 | Austria/ English | Determinants of physician's office visits and potential effects of co-payments:Evidence from Austria | quantitative/  statistic | hypothetical co-payments in the range of €5 to €200 | average annual numbers of physician’s office visits | negative impact of income and family connectedness on doctor’s visits. age, morbidity and active communication behavior in the waiting room are positively associated with office visits. | High  quality |
| 1. [62] | Herr A & Suppliet M.. 2017 | Germany/ English | Tiered co-payments, pricing, and demand in p-  for pharmaceuticals | quantitative/  statistic | price-related co-payment tiers/  exempt from co-payments | Decreasing drug prices and demand | price-related co-payment tiers are an effective tool to direct demand to low-priced drugs. | Moderate  quality |
| 1. [45] | Huber CA, et al 2012 | Germany and Switzerland/  English | Effects of cost sharing on seeking outpatient care: A propensity-matched study in Germany and Switzerland | quantitative/  statistic | introduction of (additional) cost-sharing | number of visits to a general practitioner or a specialist during the past 12 months & socio-demographic factors | significant association between health insurance scheme and the use of outpatient services. German insurant without cost sharing consulted a general practitioner or specialist more frequently than Swiss insurants with cost sharing | High  quality |
| 1. [63] | Jakobsson N & Svensson M. 2016 | Sweden/ English | Copayments and physicians’ visits: A panel data study of Swedish regions 2003-2012. | quantitative/  statistic | variation of copayments per primary care physician visit | the number of visits per capita per year | changes in the level of copayments do not affect the number of primary care physician visits | High  quality |
| 1. [64] | Jakobsson N & Svensson M. 2016 | Sweden/ English | The effect of copayments on primary care utilization: Results from a quasi-experiment. Applied | quantitative/  statistic | price reform/ co-payments in a tax-financed  health-care system | number of daily visits, socio-economic/demographic | no effect on health-care utilization and no evidence of moral hazard in the demand for GP visits. | High  quality |
| 1. [35] | Kan M & Suzuki W: 2010 | Japan/ English | Effects of cost sharing on the demand for physician services in japan: Evidence from a natural experiment | quantitative/  statistic | cost sharing: the increase in the coinsurance rate | Number of physician visits  & expenditure per visit | negative effects on physician visits,  but after the transitory period turn to be positive, negative effects on expenditures per visit that were retained even after the transitory period | High  quality |
| 1. [47] | Kiil A & Houlberg K. 2014 | Denmark/ English | How does copayment for health care services affect demand, health and redistribution? A systematic review of the empirical evidence from 1990 to 2011 | Review article | copayment | demand effects:  prescription medicine, consultations  with general practitioners and specialists, ambulatory  care and, prevalence of hospitalization | Reduction in the use of prescription medicine, consultations with GP and specialists, ambulatory care, and no significant  effects on the prevalence of hospitalisations, | High  quality |
| 1. [22] | Kim J et al 2005 | South Korea/ English | The effects of patient cost sharing on ambulatory utilization in south Korea. | quantitative/  statistic | To increase cost sharing | demand for physician service and price elasticities | out-of-pocket price elasticities depending on patient income levels and types of care setting., the users of general hospitals are less sensitive to cost sharing than the users of clinics. | High  quality |
| 1. [53] | Koc C 2011 | United States of America/ English | Disease-specific moral hazard and optimal health insurance design for physician services. | quantitative/  statistic | differential cost sharing based on disease status | optimal insurance for physician services | optimal health insurance should be designed to have differential cost  sharing based on disease status rather than to have uniform cost sharing. | High  quality |
| 1. [46] | Kullgren JT 2013 | United States of America/ English | Are the Healthy Behaviors of US High-Deductible Health  Plan Enrollees Driven by People Who Chose These Plans?  Smoking as a Case Study | quantitative/  statistic | high-deductible health plan (HDHP) | Self-reported smoking status | HDHP enrollment is associated with lower odds of smoking only among individuals who chose to enroll in an HDHP. | High  quality |
| 1. [65] | Landsem MM & Magnussen J. 2018 | Norway/ English | The effect of copayments on the utilization of the GP service in Norway | quantitative/  statistic | introduction of a co-payment | total utilization of the GP service  and this effect varies across different patient groups | overall reduction of GP visits, low price sensitivity in patients with an acute condition, strongly react in patients with general complaints and symptoms, chronic diseases and psychological diseases | High  quality |
| 1. [23] | Law CK& Yip PS. 2002 | Hong Kong/ English | Acute care service utilisation and the possible impacts of a user-fee policy in Hong Kong | quantitative/  statistic | user-fee policy | non-emergency attendances in Hong Kong | as a deterrent by preventing unnecessary use of accident and emergency services. | High  quality |
| 1. [66] | Law MR, et al (2017 | Canada/ English | Impact of income-based deductibles on drug use and health care utilization among older adults | quantitative/  statistic | The income-based deductible in BC’s Pharma Care  plan | Drug and health care utilization and cost among older adults. | A modest income based  deductible had only a trivial impact on overall access to medicines and use of other health services and may safely  reduce public spending on drugs for some population groups. | High  quality |
| 1. [67] | Lin H, Sacks DW. 2019 | United States of America/ English | Intertemporal substitution in health care demand: Evidence from the rand health insurance experiment. | quantitative/  statistic | nonlinear cost-sharing( high deductible health plan | health care demand | There is evidence of intertemporal substitution that leads to stock up on health care when it goes on sale. At the beginning of a coverage year Spending is higher in the free care plan, but at the end of a coverage year, spending in high deductible plans is slightly higher than in free care.  Short-term price changes are twice as long-term changes | High  quality |
| 1. [68] | Martinon P, et al 2018 | France/ English | On the design of optimal health insurance contracts under ex post moral hazard | Theoretical approach based on assumptions of a model | deductible | optimal health insurance | optimal contract provides includes deductible  and it may also include an upper limit on coverage. an upper limit on out-of-pocket expenses/. | moderate  quality |
| 1. [69] | Mehta N, et al 2017 | United States of America/ English | A dynamic model of health insurance choices and healthcare consumption decisions. | quantitative/  statistic | changing cost-sharing and providing more accurate information to consumers via secondary preventive care | Moral hazard (to choose more expensive medical care | incentivize consumers to purchase more secondary preventive care is more feasible  way for decreasing moral hazard | High  quality |
| 1. [15] | Mirian I et al 2020 | Iran/  English | Deductibles in health insurance, beneficial or detrimental: A review article. | review article | Deductible | -Impacts on utilization of the insured  -Financial impacts on the insured  -Financial impacts on health insurance organization | The most important positive: decrease in utilization of different services, high profitability for the young and healthy people, lower health benefit claims by the insured people, and in-crease in financial profitability of health insurance organization. and the most negative impacts increase in out-of-pocket burdens higher hospitalization | Moderate  quality |
| 1. [36] | Mortensen K.. 2010 | United States of America/ English | Copayments did not reduce Medicaid enrollees' nonemergency use of emergency departments | quantitative/  statistic | Copayments | nonemergency visits in Emergency Departments | copayments did not decrease nonemergency visits in emergency department use by  Medicaid enrollees. | High  quality |
| 1. [70] | O'Brien GL, et al 2020 | Irland /English | Out of pocket or out of control: A qualitative analysis of healthcare professional stakeholder involvement in n pharmaceutical policy change in Ireland | qualitative study/  Framework Approach | Mandatory co-payments attached to prescription medicines | the perspectives of community pharmacists and general practitioners (GPs) | Both community pharmacists and GPs accepted the theoretical concept of a co-payment on the GMS scheme as it prevents moral hazard. | High  quality |
| 1. [29] | Pauly MV& Blavin FE 2008 | United States of America/ English | Moral hazard in insurance, value-based cost sharing,  and the benefits of blissful ignorance | Theoretical approach | Value based cost sharing | Optimal insurance | if patient demands  are based on correct information,. otherwise  optimal coinsurance depends both on information imperfection and price responsiveness. Therefore Value-based cost sharing can be taking precedence over to providing information | Moderate  quality |
| 1. [48] | Petrou P  2015 | Cyprus/ English | An interrupted time-series analysis to assess impact of introduction of co-payment on emergency room visits in Cyprus/ | quantitative/  statistic | introduction of co-payment fee of EUR10 | emergency  room services. | reduction of emergency room visits. Nom impact was observed in categories of teenagers, children, infants, and people over 70 years old. | High  quality |
| 1. [37] | Pütz C& Hagist C  2006 | Germany/ English | Optional deductibles in social health insurance systems: Findings from Germany. | quantitative/  statistic | bonus of €240 per year plus to pay a deductible for their medical treatment of up to €300. | - compatible with the principles of solidarity;  -insurance  claims (moral hazard). | compatible with the principles of solidarity  and reduction of insurance  claims (moral hazard) | Moderate  quality |
| 1. [71] | Rabin et al  2020 | United States of America/ English | Under the aca higher deductibles and medical debt cause those most vulnerable to defer needed care. | quantitative/  statistic | Deductibles  increased in employer- provided insurance, combine HRAs with HDHPs. | medical debt,  deferred needed care | Rates of medical debt and deferred care decreased, Medical debt and deferred needed medical care is greater for vulnerable group(lower income, minority, treatable chronic diseases). | High  quality |
| 1. [52] | Reddy SR, et al (2014) | United States of America/ English | Impact of a high-deductible health plan on outpatient visits and associated diagnostic test | quantitative/  statistic | High-Deductible Health Plan (HDHP) | Outpatient Visits and Associated Diagnostic Tests  laboratory and radiology tests | - Moderate reduction in overall office visits - no significant differences in changes in visit rates for acute higher or lower-priority conditions - moderate relative reductions in the use of general laboratory tests but not in radiology tests. | moderate  quality |
| 1. [24] | Reichmann G, et al 2004 | Austria/ English | Co-payments in the Austrian social health insurance system - analysing patient behaviour and patients' views on the effects of co-payments | quantitative/  statistic | introduction of additional, mainly lump sum co-payments and the increase of existing (lump sum) co-payments | patients’ attitudes, their views on the  effects of co-payments on health care demand and their actual behaviour in response to co-payments | co-payments have no major effect on health care demand that confirmed by what the patients indicate as regards their actual behaviour. | High  quality |
| 1. [72] | Sabik LM & Gandhi SO. 2016 | United States of America/ English | Copayments and emergency department use among adult Medicaid enrollees. | quantitative/  statistic | changes in Medicaid ED copayment policies (increase copayment) | nonurgent Emergency department ED utilization among  nonelderly adult enrollees | visits among Medicaid enrollees are significantly less | Moderate  quality |
| 1. [25] | Schellhorn, M. (2001). | Swiss/ English | The effect of variable health insurance deductibles on the demand for physician visits. | quantitative / statistic | introduction of a choice of  deductible  for health services in the mandatory basic health insurance | reduce moral hazard:physician service utilization. | no significant effect of such a choice on utilization | Moderate  quality |
| 1. [26] | Schreyögg J.  2004 | Germany / English Singapore,  South Africa, China and the U.S. | Demographic development and moral hazard: Health insurance with medical savings accounts | qualitative study (comparative) | Medical Savings Accounts | Objective,  Enrolment,  Financing form of high-risk insurance,  Administration,  Service areas,  Coverage of population, Annual interest payment | it could be feasible to integrate certain elements of this concept into health care systems of European countries | Moderate  quality |
| 1. [6] | Schreyogg J& Grabka MM 2010 | Germany/  English | Copayments for ambulatory care in Germany: a natural experiment using a difference-in-difference approach | quantitative/  statistic | introduction copayment for ambulatory care in 2004 for individuals with statutory health insurance | overall demand for physician visits | no significant reduction in  the number of physician visits among SHI members compared to control group, and no deterrent effect among vulnerable individuals. | High  quality |
| 1. [5] | Schubert S.  2014 | Germany/  English | Reducing public health insurance expenditure: A numerical analysis for Germany | quantitative/  statistic | mandatory deductibles and further elevating copayments | health care  demand:  health care expenditure | very small decrease in expenditure associated with both reform options | Moderate  quality |
| 1. [78] | Serna N. 2021 | United States of America/ English | Cost sharing and the demand for health services in a regulated market. | Quantitative: | tier coinsurance and income base copays | utilization of health services | Reduction in elective and preventive services with higher cost sharing | High  quality |
| 1. [49] | Sinnott SJ, et al 2013 | Ireland/  English | 'What is 50cent?' - a qualitative study of patient attitudes and medicine taking behaviours in response to a 50cent charge on prescription drugs in a publicly funded health system in Ireland. | qualitative study/  Framework method | 50 cent the price of copayment with capped at €10 per  household monthly | participant opinions and insights relate to  how symbolic copayments could play a role in reducing moral hazard,, without implicating patient adherence to important medicines | conflicting opinion on whether 50 cent can stop moral hazard | High  quality |
| 1. [50] | Steinorth P. J 2011 | Germany/ English | Impact of health savings accounts on precautionary savings, demand for health insurance and prevention effort | theoretical approach based on assumptions of a model | health savings accounts | optimal savings,  insurance demand and prevention effort over the course of a lifetime | .an increased tax subsidy may worsen moral hazard for prevention efforts and precautionary savings, it is stated that consumers do not take these two measures at the same time, in case of precautionary savings, preventive action is reduced, and vice versa | Moderate  quality |
| 1. [9] | Trottmann M, et al 2012 | Switzerland/  English | Supply-side and demand-side cost sharing in deregulated social health insurance: Which is more effective? | Quantitative/ statistic | Supply-side cost sharing and demand-side cost sharing (through voluntary deductibles) | effective in curtailing the use of medical services | both types of cost sharing to be effective in curtailing the  use of medical services, however, supply-side cost sharing option is more effective | High  quality |
| 1. [27] | Ullrich CG 2002 | Germany/ English | Managing the behavior of the medically insured in Germany: The acceptance of cost-sharing and risk premiums by members of the statutory health insurance | qualitative guided interviews | cost-sharing and risk premiums | social acceptance of cost-sharing and risk premiums by members of the German statutory health insurance. | controlling consumptive behavior through cost sharing  are much more optimistically and Management of behavior by cost sharing is considered more often to be justified. | Moderate  quality |
| 1. [38] | van Kleef RC, et al2009 | Netherlands/ English | Shifted deductibles for high risks: More effective in reducing moral hazard than traditional deductibles | quantitative/  statistic | Shifted  Deductibles | moral hazard | shifted deductibles with a risk-adjusted starting point  leads lower out-of-pocket expenditures and may further reduce moral hazard. | High  quality |
| 1. [51] | van Winssen KP  2015 | Netherlands/ English | How profitable is a voluntary deductible in health insurance for the consumer? | quantitative/  statistic | voluntary deductible (VD) in return for a premium rebate. | financial profitability | is profitable for males, young insured, healthy insured and insured with few healthcare expenses in the past. | High  quality |
| 1. [28] | Winkelmann R. 2004 | Germany/ English | Co-payments for prescription drugs and the demand for doctor visits - evidence from a natural experiment. | quantitative/  statistic | To increase co-payments for prescription drugs | price sensitivity of demand  for physicians’ services | increased co-payments reduced the number of doctor visits | High  quality |
| 1. [79] | Wu Y etal . 2021; | China/ English | Health care insurance policies when the provider and patient may collude | Theoretical approach based on assumptions of a model | Placing the incentive on the patient (risk premium)&provider | Social cost | Placing the incentive on the patient is less social costly than placing the incentive on the provider if the risk premium of the patient to escape the insurance's imperfect pure risk is  smaller than the informational rent given to the provider. | Moderate  quality |
| 1. [73] | Yoo KB, et al. 2016 | Korea/ English g | Impact of co-payment for outpatient utilization among medical aid beneficiaries in Korea: | quantitative/  statistic | introduction of out- patient co-payment scheme. | medical cost,  out patients and inpatients visits | increasing inpatient utilization and medical cost | High  quality |
| 1. [74] | Zhang H & Yuen P 2016 | China/ English | Medical savings account balance and outpatient utilization: Evidence from Guangzhou, china | quantitative/  statistic | Medical Savings Account balance | outpatient utilization | negative relationship between MSA balance and outpatient utilizationis, usefulness of MSA for“Cost-containment”, “Savings for the future” andEnabling utilization”. | High  quality |
