# Supplementary material for: A systematic review of strategies used for controlling consumer moral hazard in health systems
Source: BMC Health Serv Res. 2022 Oct 18;22:1260. doi: 10.1186/s12913-022-08613-y (PMC9580205; doi:10.1186/s12913-022-08613-y)
Supplement: Supplementary file 3 — Supplementary Material 3 [file 12913_2022_8613_MOESM3_ESM.docx]

| **Code**  Additional fil3: The sources from which each code is extracted | **References** |
| --- | --- |
| 1. deductible 2. traditional deductible 3. mandatory deductible 4. First euro deductible/ a first-dollar deductible. 5. doughnut hole deductible (a gap in coverage after paying a fixed amount of healthcare expenses) 6. co-payments**/**copayment, flat/fix copayment / user charges/ user-fee policy / lump sum co-payments, fixed payment/ Mandatory co-payments 7. coinsurance/co-insurance 8. limit on coverage caps on insurance /stop loss. / payment ceiling | 1. (9, 10, 15, 19, 68) 2. (38) 3. (5) 4. (57) 5. (57) 6. (6, 9, 10, 20, 22-24, 28, 31, 32, 36, 42, 45, 47, 48, 61, 63-65, 70, 72, 73, 93) 7. (30, 31, 35, 43) 8. (9, 10, 68) |
| 1. Shift deductible 2. Income base deductible 3. variable health insurance deductible 4. optional deductible (rates incorporating deductibles) 5. voluntary deductible (VD) 6. high‐deductible health plans (HDHPs), nonlinear cost-sharing 7. consumer‐directed health plans (CDHPs) /Consumer-Driven Health Plans (CDHPs) /Consumer engagement/ three-tier payment system/HDHPs coupled with personal savings account 8. income-related copayment 9. multitiered copayments / tiered copayment/ price-related co-payment tier/ co-payment exemption 10. co-payment with rebate 11. co-payment with premium reduction 12. different copayment according to the socioeconomic status or disease severity 13. Value Based Insurance Design (VBID) / value-based cost sharing (lower coinsurance for services with higher costs benefits / value-based coinsurance target treatment-specific copayments /disease-specific cost sharing/ differential cost sharing based on disease status; /variable co-insurance based on demand elasticity / shared savings policy SSP/ waiving copays | 1. (38) 2. (66) 3. (25) 4. (37) 5. (51, 55) 6. (40, 46, 52, 54, 56, 60, 67, 71) 7. (40, 54, 60) 8. (39) 9. (62) 10. (42) 11. (42) 12. (41) 13. (18, 29, 44, 53) |
| 1. health savings accounts (HSAs) / medical savings accounts (MSAs) in combination with privet insurance | 1. (50, 54, 71, 74) |
| 1. medical savings accounts (MSAs) in combination with social health insurance/ medisave/medisave accounts 2. health savings accounts (HSAs) in combination with social risk-pooling (SRP) /tongdao (MSA in combination with SRF as an integrated)/ (through-path) medical savings accounts (MSAs) in combination with social insurance pool (SIP)/ the three-tiered design (MSA-deductible-SIP) 3. bankuai (MSA separately to finance outpatient services)/(compartment) | 1. (26, 74, 75) 2. (58, 74, 75) 3. (74) |
| 1. uniform price/uniform prices/ uniform pricing/uniform monopoly pricing, 2. discriminatory pricing 3. two-part tariffs (combines a uniform price with market-specific lump-sum payments) 4. third degree price discrimination/ (charging different prices to different markets or groups of consumers | 1. (21) 2. (21) 3. (21) 4. (21) |
| 1. waiting lists / /waiting list queues/ queuing / waiting time / expectancy queue /the cost of the lost time | 1. (10, 31, 34) |
| 1. proposing insurance coverage for preventive care 2. separating insurance coverage for prevention and treatment 3. to encourage insureds to use more secondary preventive care 4. Improving perception of health status through secondary preventive care | 1. (30) 2. (30) 3. (69) 4. (69) |
| 1. premiums are adjusted according level of preventive effort 2. bonuses/ bonus payments/ rebate insurance /to encourage non-use or limited use in return for next premium reduction /rebates, risk premium risk adjustment no-claims bonus 3. risk rating premium 4. No-claim Bonus and Coverage Upper Bound | 1. (30) 2. (10, 15, 27, 39, 59) 3. (76) 4. (77) |
| 1. Health promotion education 2. Civic education about the consequences of unnecessary use of health services | 1. (23) 2. (23) |
